# Changes in the incidence, viral coinfection pattern and outcomes of pneumococcal hospitalizations during and after the COVID-19 pandemic

**DOI:** 10.1186/s41479-025-00164-0

**Published:** 2025-04-25

**Authors:** King-Pui Florence Chan, Ting-Fung Ma, Hanshu Fang, Wai-Kai Tsui, James Chung-Man Ho, Mary Sau-Man Ip, Pak-Leung Ho

**Affiliations:** 1 Department of Medicine, University of Hong Kong, Queen Mary Hospital, Hong Kong SAR, China; 2 Department of Statistics, University of South Carolina, Columbia, South Carolina USA; 3 Department of Microbiology and Carol Yu Centre for Infection, Queen Mary Hospital, University of Hong Kong, Pokfulam Road, Hong Kong SAR, China

**Keywords:** Pneumococcal disease, Coinfection, Influenza, Coronavirus disease 2019

## Abstract

**Background:**

The incidence of pneumococcal pneumonia in the context of the Coronavirus Disease 2019 (COVID-19) pandemic, along with the real-world data on the ratio of non-invasive to invasive pneumococcal pneumonia, is an area that has not been thoroughly studied. The outcomes associated with coinfection of influenza and COVID-19 remain unknown. This study examined the incidence, demographics, coinfection with influenza and/or COVID-19, and clinical outcomes of pneumococcal hospitalizations in Hong Kong during the baseline, pandemic, and post-pandemic periods.

**Methods:**

Hospitalization records of individuals aged 18 years and above with pneumococcal disease from January 2015 to August 2024 were extracted from the territory-wide electronic medical record database. Pneumococcal disease was categorized into invasive pneumococcal pneumonia (IPP), invasive pneumococcal disease without pneumonia (IPDWP), and non-invasive pneumococcal pneumonia (NIPP), followed by univariate and multivariate analyses. Effects of coinfection with influenza and COVID-19 were analyzed.

**Results:**

The incidence of pneumococcal disease decreased during the COVID-19 pandemic but rebounded in the post-pandemic period. There were no significant changes in the distribution of pneumococcal serotypes across the three periods. The study revealed a strong positive correlation between monthly pneumococcal hospitalizations and the indicator of influenza activity, while the correlation with the COVID-19 indicator was weak. Additionally, strong positive correlations were observed between the indicator of influenza activity and influenza coinfections, as well as between the indicator of COVID-19 activity and COVID-19 coinfections. Multivariate analyses identified male gender, a higher comorbidity index, older age, invasive pneumococcal disease (IPP and IPDWP), coinfection with influenza and COVID-19, and hospitalization during the pandemic period as factors associated with adverse outcomes.

**Conclusions:**

The study showcases changes in the epidemiology of pneumococcal disease during and after the COVID-19 pandemic. It highlights the adverse effects of influenza and COVID-19 coinfections on the outcomes of patients with pneumococcal disease and emphasizes the need to vaccinate vulnerable populations against these infections.

**Supplementary Information:**

The online version contains supplementary material available at 10.1186/s41479-025-00164-0.

## Background

Pneumococcal disease is caused by *Streptococcal pneumoniae* infection, with at least 100 known serotypes of pneumococci [1]. Pneumococcal disease can be broadly categorized into invasive pneumococcal disease (IPD) or non-invasive pneumococcal disease, with IPD defined by the detection of *S. pneumoniae* from a normally sterile body site. The major clinical syndromes of pneumococcal disease are pneumonia, bacteremia and meningitis [2, 3]. Pneumococcal pneumonia is commonly defined as radiologically confirmed pneumonia with a test result positive for the organism [4]. Pneumococcal vaccine was shown to decrease the incidence of invasive pneumococcal disease [3, 5]. In Hong Kong, pneumococcal vaccine is recommended for all children younger than 2 years old, adults 65 years and older, and individuals with high risk predisposing conditions for pneumococcal disease [6]. In children, pneumococcal conjugate vaccine was administered routinely as part of the children immunization program since 2009, achieving a primary series and booster dose uptake rate of 97% and higher [7–9]. In a survey conducted in 2023, it is estimated that 41% of the elderly population aged 65 years or older in Hong Kong have been administered at least one dose of either the pneumococcal conjugate vaccine or the 23-valent polysaccharide vaccine [7].

The World Health Organization declared coronavirus disease 2019 (COVID-19), caused by the severe acute respiratory syndrome coronavirus 2 (SARS-CoV-2), a public health emergency of international concern on 30 January 2020 [10]. In response, universal masking and social distancing were widely practiced worldwide including in Hong Kong, leading to a documented decrease in the incidence of influenza and changes in the epidemiology pleural empyema and tuberculosis [11–14]. Our group previously reported a decrease in the incidence of invasive pneumococcal disease (IPD), pneumococcal pneumonia, and all-cause pneumonia during the COVID-19 pandemic [15]. Following the relaxation of social distancing measures by Hong Kong government on 22 December 2022, public health measures such as universal masking, social distancing, and travel restriction were lifted [16, 17]. This was followed by an increase in the incidence of influenza in Hong Kong amidst the on-going COVID-19 infection [18, 19].

The objective of this study is to investigate changes in the incidence rate and clinical outcomes of pneumococcal hospitalizations in Hong Kong during and after the COVID-19 pandemic. Additionally, we analyzed the temporal trends of coinfection with influenza and COVID-19.

## Methods

### Study design

A retrospective observational cohort study was conducted in Hong Kong using territory-wide data from the Hospital Authority, the statutory body overseeing all public hospitals, and providing 90% of in-patient services to the city’s 7.5 million population [14]. The study examined adult patients (aged ≥ 18 years) hospitalized with pneumococcal disease from January 2015 to August 2024, segmented into baseline (January 2015 to December 2019), COVID-19 pandemic (January 2020 to December 2022), and post-pandemic (January 2023 to August 2024) periods for analysis.

### Data source

Data based on episodes was extracted from the Hospital Authority's territory-wide electronic medical record database, Clinical Data Analysis and Reporting System (CDARS), where patient identities were anonymized [14]. Medical records of patients hospitalized for pneumococcal disease were accessed using ICD-9 codes for pneumococcal disease, including 028.2 (pneumococcal septicemia), 041.2 (pneumococcal infection), 320 (pneumococcal meningitis), and 418 (pneumococcal pneumonia), as well as positive pneumococcal test results (culture, polymerase chain reaction, and pneumococcal urinary test results). The data collected for analysis included patient demographics, clinical details, and microbiological information.

Monthly number of severe COVID-19 cases was collected from a surveillance conducted by the Centre for Health Protection (CHP) of the Department of Health, converted to an incidence rate (per 10,000 population), and used as an indicator of COVID-19 activity. Monthly percentage of influenza positivity in respiratory specimens was collected from the CHP’s laboratory surveillance for both inpatients and outpatients in both public and private medical sectors and used as an indicator for influenza activity. Influenza season was delineated as the time period when influenza positivity percentage in respiratory samples was above the threshold of 9.21% and influenza-associated hospital admission rate was above the threshold of 0.25 cases per 10,000 population [20, 21].

Invasive pneumococcal disease (IPD) was defined as the first hospitalization per person with (1) a discharge diagnosis of pneumococcal meningitis (ICD-9 code 320.1), pneumococcal septicemia/bacteremia (ICD-9 code 028.2), or pneumococcal infection (ICD-9 code 041.2) involving a normally sterile site, pleural empyema (ICD-9 code 510.9), septic arthritis (ICD-9 code 711.), spontaneous bacterial peritonitis (ICD-9 code 567.23), endophthalmitis (ICD-9 code 360.00); and/or (2) positive culture and/or PCR detection of pneumococcus in a normally sterile site. These sites included blood, cerebrospinal fluid, pleural fluid, peritoneal fluid, joint fluid, and internal body sites. Cases of IPD were categorized into invasive pneumococcal pneumonia (IPP) and invasive pneumococcal disease without pneumonia (IPDWP) based on the presence or absence of pneumonia. Non-invasive pneumococcal pneumonia (NIPP) was defined as the first hospitalization per person with (1) a discharge diagnosis of pneumococcal pneumonia (ICD-9 code 481) or (2) pneumonia (ICD-9 codes 482.30, 486) with a positive pneumococcal urinary antigen test (UAT) and/or positive culture of pneumococcus from lower respiratory tract specimens, and negative laboratory confirmation for pneumococcus from normally sterile sites and absence of discharge diagnosis for IPD. Lower respiratory tract specimens included sputum, tracheal aspirate, bronchial aspirate and bronchoalveolar lavage. Coinfection of pneumococcal disease with COVID-19 or influenza was defined as positive PCR results for SARS-CoV-2 or influenza, respectively, within 15 days before or after the date of admission [14]. The serotypes of the isolates were determined by multiplex PCR assays, which covered 35 serotypes including all PCV13 serotypes, and latex agglutination tests in two reference laboratories [22]. Nontypeable isolate was further analysed by next generation sequencing [23, 24].

### Inclusion and exclusion criteria

Patients admitted to an acute hospital and then transferred to convalescent hospitals within the same hospitalization were considered as a single episode. Hospitalization episodes for pneumonia or other infections in which the pneumococcal etiology was not documented by pneumococcal ICD-9 coding and/or positive pneumococcal test results were excluded. These exclusions included pneumonia episodes with the detection of pneumococcus in throat swab, nasal swab, or nasopharyngeal swab only; pneumonia of unspecified etiology (ICD-9 code 486) where laboratory verification of pneumococcal infection is absent; and instances where urinary antigen testing and/or culture from lower respiratory specimens were positive but there was no corresponding pneumonia coding.

### Outcome

The study aimed to compare outcomes across three periods: baseline, COVID-19 pandemic, and post-pandemic periods. The primary outcome was episode death, defined as death in the index admission. Secondary outcomes included hospital length of stay, the need for intensive care unit (ICU) admission, and invasive mechanical ventilation (IMV).

### Statistical analysis

The incidence, demographic, and clinical data were presented in actual frequency, proportion, and median (interquartile range, IQR) as appropriate [11, 12, 15]. To compare data across the baseline, COVID-19 pandemic, and post-pandemic periods, the Mann–Whitney U test was used for continuous variables, and the Chi-square test was used for categorical variables. The annual incidence rate for each pneumococcal disease was computed using the entire population, categorized by age groups (18–59 years, 59–74 years, and ≥ 75 years), and expressed as per 100,000 person-years. Age-specific population data were sourced from the Census and Statistics Department.

The change in the incidence rate of hospitalizations across the three time periods was estimated as in our previous study [15]. Spearman's rank correlation coefficient was used to assessed the association between the indicators of COVID-19 or influenza activity in Hong Kong and pneumococcal hospitalizations, as well as coinfection with COVID-19 and influenza [25]. Cumulative episode deaths between the baseline, COVID-19, and post-pandemic periods were compared using the log-rank test to detect any statistical differences between the baseline and pandemic periods, as well as between the baseline and post-pandemic periods [26].

Multivariate logistic regression models were used to investigate the binary outcomes and independent variables, namely age group (18–59 years, 60–69 years or ≥ 70 years), sex, Charlson Comorbidity Index (CCI), type of diseases (IPP, IPDWP, NIPP), pleural empyema, time periods (baseline, COVID-19 pandemic or post-pandemic), and coinfection with influenza or COVID-19. For age group, 18–59 years was used as reference against other age groups. CCI was classified as two categories (< 4 and ≥ 4 points) making reference to the median CCI score in the cohort. NIPP was used as reference group for type of pneumococcal disease. All data and outcomes were censored on 31 August 2024. Two sensitivity analyses were performed using Log-binominal regression and robust Poisson regression. A *P* value of < 0.05 was considered to indicate statistical significance. Adjusted odds ratio (OR), adjusted risk ratio (RR) and 95% confidence interval (CI) were calculated for three outcomes (episode death, ICU admission, IMV use) in the entire cohort.

## Results

### Patient demographics

A total of 5,517 hospitalizations for pneumococcal infections, including 920 cases of IPD and 4,597 cases of NIPP, were analyzed (Table 1 and Figure S1). Across the three time periods, variations were observed in the genders of patients (male 55.2%−68.4%, female 31.6%−44.8), elderly individuals aged ≥ 70 years (52.5%−73.9%), and the presence of underlying conditions such as diabetes mellitus, stroke, renal disease, chronic pulmonary disease, and congestive heart failure.

**Table 1:** Characteristics of patient hospitalized pneumococcal infections

Variable^a^	Baseline (*n* = 3495)	COVID-19 pandemic (*n* = 1014)	Post- Pandemic (*n* = 1008)	*P*
Male	68.4 (2389)	60.0 (608)	55.2 (556)	< 0.005
Age, median (IQR), y	71 (60–82)	80 (68–89)	77 (65–88)	< 0.005
Age groups				
18–59 years	24.4 (852)	12.4 (126)	17.8 (179)	< 0.005
60–69 years	23.1 (807)	13.7 (139)	15.9 (160)	< 0.005
≥ 70 years	52.5 (1836)	73.9 (749)	66.4 (669)	< 0.005
Comorbidity, % (no.)				
Any (at least one)	54.6 (1909)	70.8 (718)	64.0 (645)	< 0.005
Diabetes mellitus	14.3 (500)	23.2 (235)	25.3 (255)	< 0.005
Liver disease	5.0 (176)	3.9 (40)	5.9 (59)	0.137
Stroke	11.8 (412)	26.1 (265)	18.0 (181)	< 0.005
Malignancy	10.2 (357)	12.6 (128)	12.0 (121)	0.050
Renal disease	4.9 (173)	9.6 (97)	7.7 (78)	< 0.005
Chronic pulmonary disease	14.6 (511)	13.2 (134)	9.9 (100)	< 0.005
Congestive heart failure	8.0 (280)	13.5 (137)	12.4 (125)	< 0.005
Rheumatological disease	1.9 (67)	1.8 (18)	1.4 (14)	0.540
HIV infection	0.4 (14)	0.6 (6)	0.6 (6)	0.600
Charlson comorbidity index ≥ 4	53.0 (1852)	75.3 (764)	66.4 (669)	< 0.005
Type of pneumococcal disease				
IPDWP	7.4 (259)	3.6 (37)	4.4 (44)	< 0.005
IPP	12.9 (451)	4.7 (48)	8.0 (81)	< 0.005
NIPP	79.7 (2785)	91.6 (929)	87.6 (883)	< 0.005
Pneumococcal disease by site				
Bacteremia	17.3 (603)	6.8 (69)	10.1 (102)	< 0.005
Pneumonia	92.6 (3236)	96.4 (977)	95.6 (964)	< 0.005
Pleural empyema	2.2 (78)	1.0 (10)	2.0 (20)	0.040
Meningitis	1.9 (66)	1.2 (12)	1.4 (14)	0.227
Viral coinfection				
Influenza	8.8 (309)	1.2 (12)	12.1 (122)	< 0.005
COVID-19	-	9.0 (91)	10.9 (110)	0.987

*Abbreviations*: *HIV* human immunodeficiency virus, *IPDWP* invasive pneumococcal disease without pneumonia, *IPP* invasive pneumococcal pneumonia, *IQR* interquartile range, *NIPP* non-invasive pneumococcal pneumonia, *y* year

^a^Values indicate % (number) by column unless otherwise indicated

Of the 920 IPD cases, 361 cases were identified through positive test results from a sterile site, 264 cases were identified through diagnostic coding, and 295 cases were identified through both test results and coding. In the 264 cases identified through diagnostic coding, 158 cases had either positive UAT or a positive sputum culture for *S. pneumoniae*. The majority had isolates recovered from blood cultures (62.3%). Other culture-positive sites included CSF (4.9%), pleural fluid (3.4%), joint fluid (0.9%), ocular fluid (0.7%), and peritoneal fluid (0.3%). Additionally, 10 cases had CSF positive by PCR. Among the IPD cases, 63% were classified as IPP, while 37% were categorized as IPDWP. Of the 4597 NIPP cases, 7.4% had positive pneumococcal cultures from lower respiratory tract specimens, 58.1% had positive UAT results, and 0.7% had positive pneumococcal results from both types of tests. The remaining 36.6% NIPP cases were identified through pneumococcal coding. The proportions of IPDWP, IPP, and NIPP across the time periods, were 3.6%−7.4%, 4.7%−12.9% and 79.7%- 91.6%, respectively (Table 1). The ratio of NIPP to IPP ranged from 5.0–8.0 in baseline, 16.5–21 in COVID-19 and 10.2–12 in post-pandemic period. These cases included 1.0%−2.2% with pleural empyema and 1.2%−1.9% with meningitis. Coinfection rates of 1.2%−12.1% and 9.0%−10.9% were observed for influenza and COVID-19, respectively (Table 1). In 93.2% and 92.5% of the coinfection cases, positive test results for influenza and COVID-19 were detected on the day of admission or the following day.

### Changes in incidence rate by time periods

**Fig. 1:**
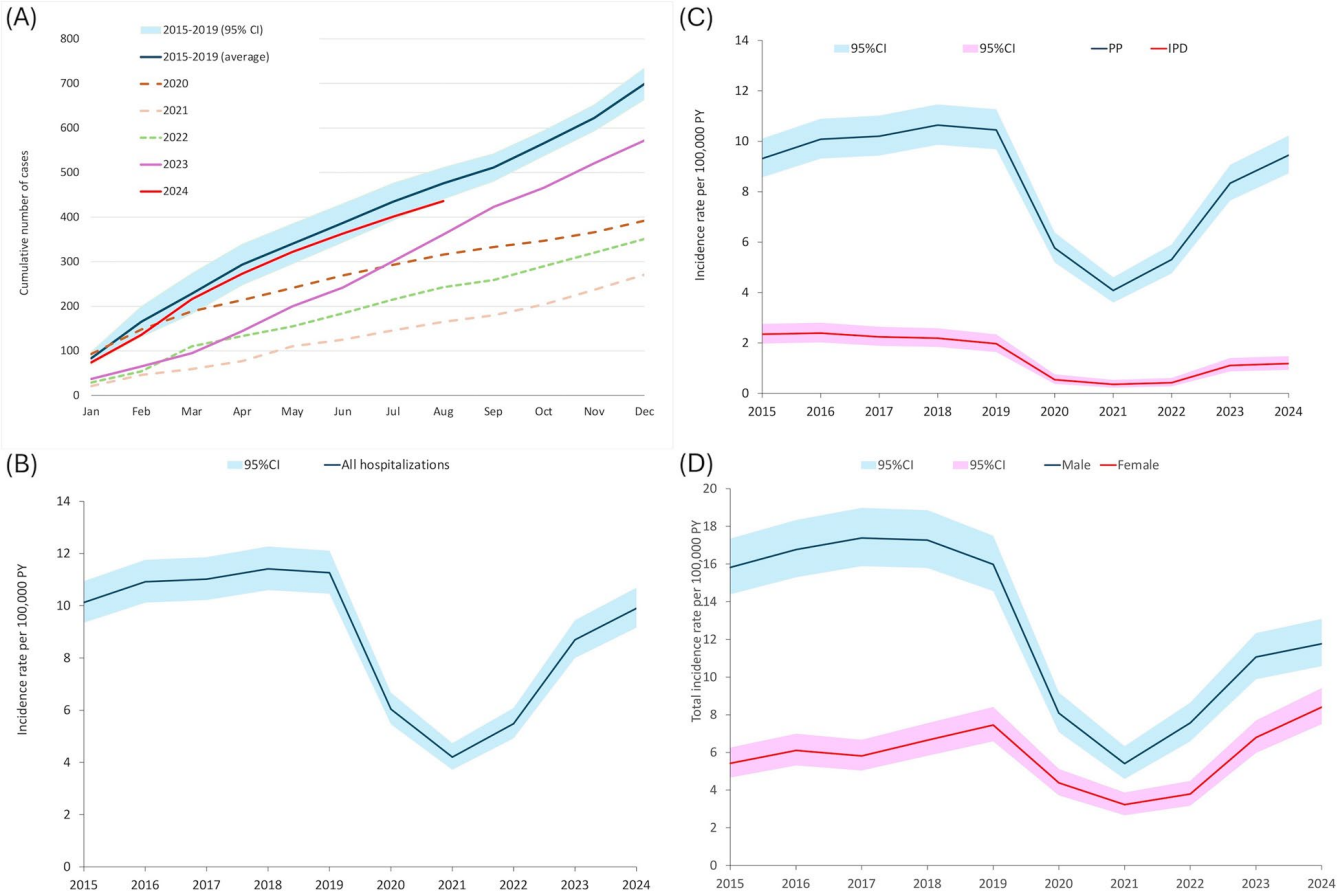
Temporal changes in number and incidence rate of pneumococcal hospitalizations, 2015–2024. **A** Cumulative number (**B**) annual incidence rate (**C**) incidence rate for invasive pneumococcal disease (IPD) and pneumococcal pneumonia (PP). IPD included invasive disease with and without pneumonia. PP included both invasive and non-invasive pneumonia. **D** incidence rate by gender

The number of pneumococcal hospitalizations and incidence rate (cases per 100,000 person years (PY)) declined substantially in 2020–2022 but gradually increased since March 2023 (Fig. 1A and B). The annual number (572 cases) and incidence rate (8.7, [95% CI 8.0–9.4] cases per 100,000 PY) in 2023 was higher than that in 2020–2022 (271–392 cases and 4.2–6.0 cases per 100,000 PY) but remained lower than baseline average for year 2015–2019 (699 cases and 11.0 [95% CI 10.6–11.3] cases per 100,000 PY). In 2024, the estimated annual number and incidence rate (654 cases and 9.9 [95% CI 9.2–10.70] cases per 100,000 PY) were similar to the range during baseline years. A similar change in trend was observed after stratification by type of pneumococcal disease and patient gender (Fig. 1C and D). Additionally, comparable temporal changes in the incidence rates were observed for both laboratory-confirmed cases and the pneumococcal ICD-coding cases (Figure S2).

### Temporal changes in serotype distribution

**Fig. 2:**
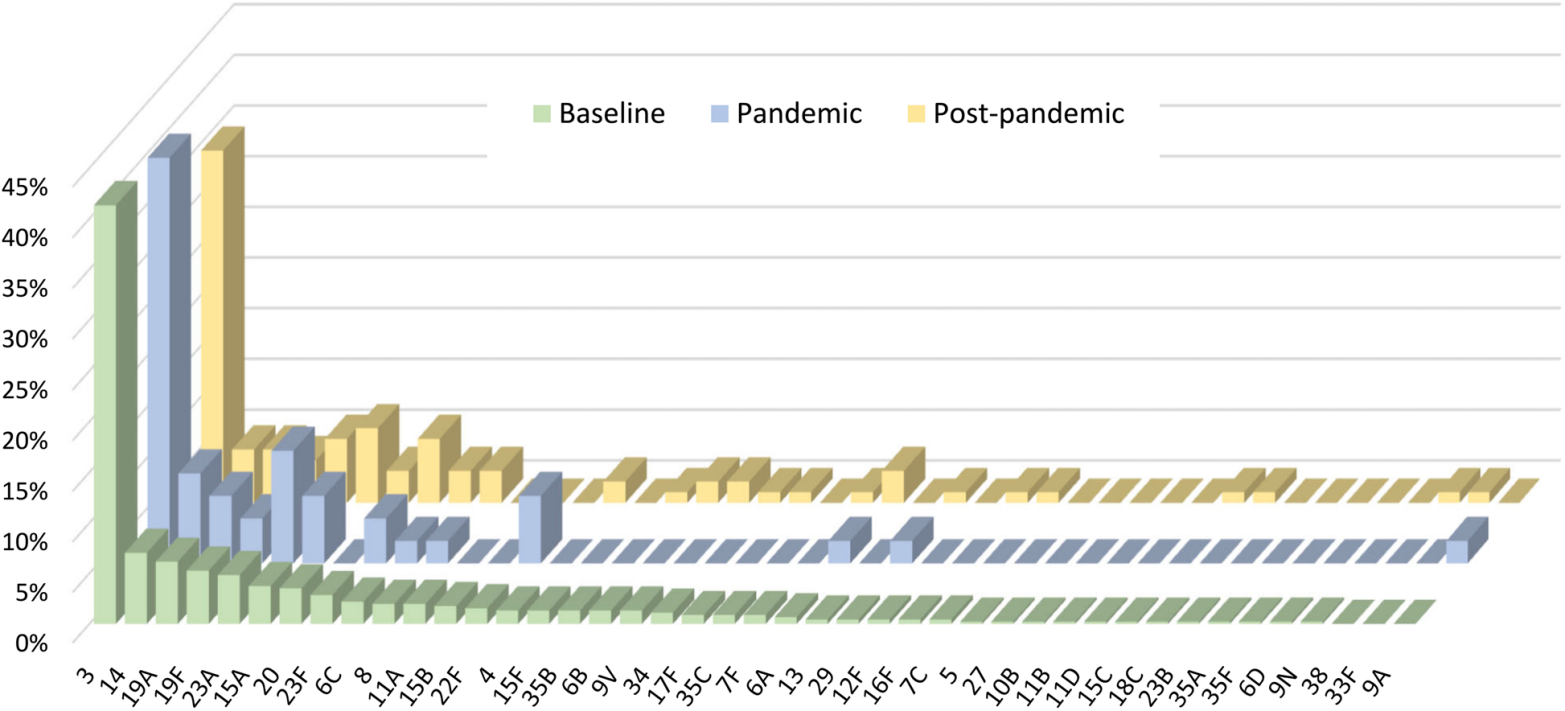
Distribution of pneumococcal serotypes in by time periods

Serotype information was available for 595 cases, including 189 IPDWP, 323 IPP, and 83 NIPP cases (Fig. 2). No significant changes in the distribution of serotypes were observed. In the baseline, pandemic, and post-pandemic periods, the proportions (95% confidence interval) of serotypes covered in PCV15 were 63.1% (55.0%−70.8%), 64.4% (43.2%−92.6%), and 63.2% (48.2%−81.3%), respectively. Additional serotypes covered by PCV20 (including 8, 10A, 11A, 12F, 15B) accounted for 5.4% of the overall isolates, ranging from 2.2% to 6.2% during the three periods. Serotype 3 was the most prevalent, making up 34.7% to 41.3% of all serotypes across the three periods. Other common serotypes (> 3% frequency) were 14, 19A, 19F, 23A, 15A, 20, and 23F. The mortality of serotype 3 was 23.0% compared to 22.2% of non-serotype 3 (*P* = 0.814) but it was associated with a higher rate of ICU admission (25.9% vs 15.7%, *P* = 0.003).

### Temporal changes in coinfection with influenza and COVID-19

**Fig. 3:**
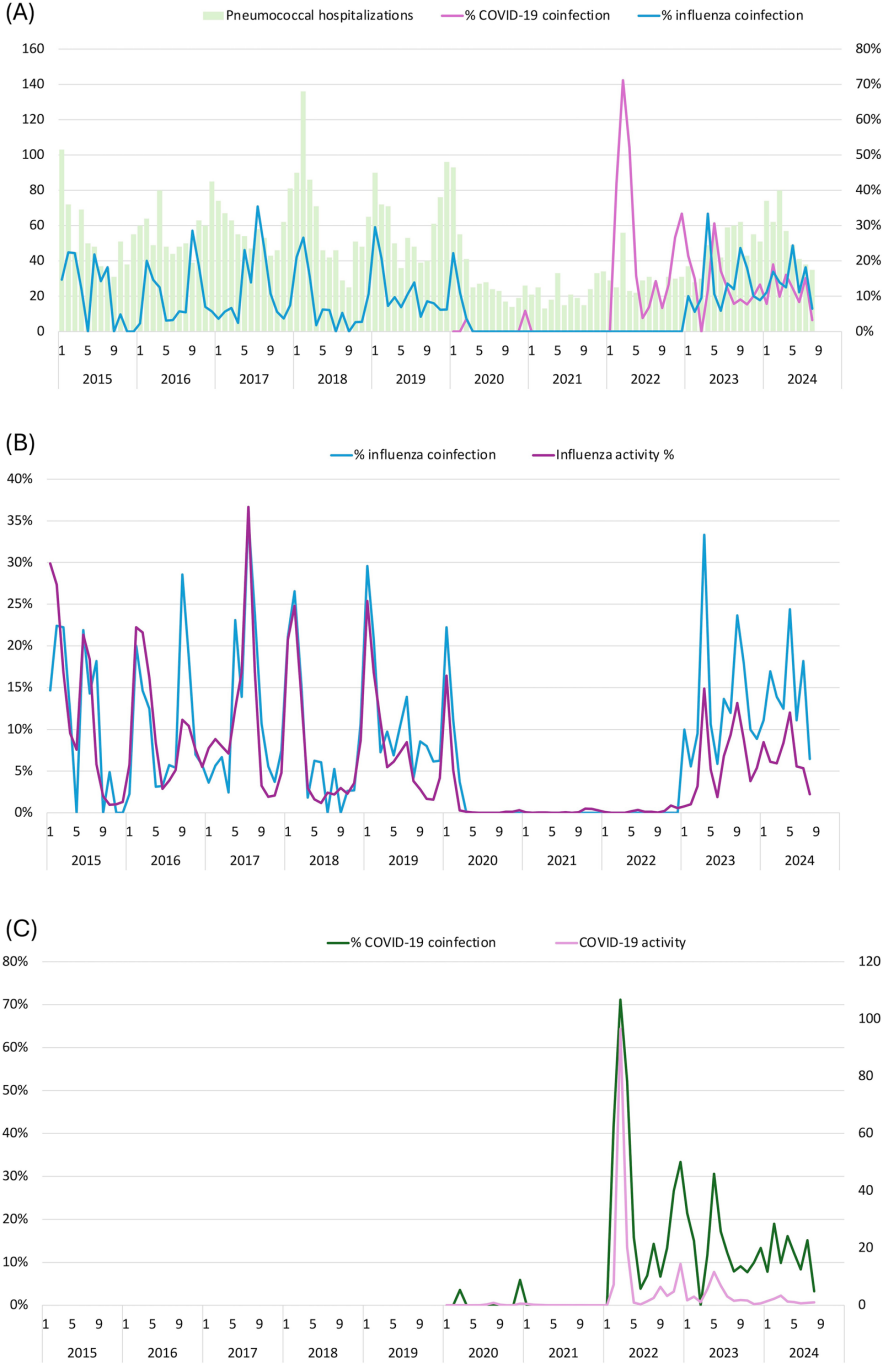
Pneumococcal hospitalization and coinfection with influenza and COVID-19, January 2015- August 2024. **A** monthly pneumococcal hospitalization and percentages with influenza and COVID-19 coinfection, Spearman’s rank correlation coefficient of influenza activity with monthly pneumococcal hospitalization = 0.755, *P* < 0.001, COVID-19 activity with monthly pneumococcal hospitalization = 0.755, *P* < 0.001 (**B**) percentages of influenza coinfection and indicator of influenza activity, Spearman’s rank correlation coefficient = 0.876, *P* < 0.001 (**C**) percentages of COVID-19 coinfection and indicator of COVID-19 activity, Spearman’s rank correlation coefficient = 0.847, *P* < 0.001

During the study period, a total of 12 influenza seasons were documented (Table 2 and Fig. 3A), encompassing 6 winter seasons, 3 spring seasons, 2 summer seasons, and 1 autumn season. The 2019/20 season concluded prematurely in early February, shortly after the widespread adoption of community mask-wearing practices. Subsequently, no influenza season was observed until March 2023. The percentage of cases tested for influenza during the baseline (76.6%) and post-pandemic (84.6%) periods was higher than during the pandemic period (37.0%), which was characterized by very low influenza activity (Figure S3). In the baseline period, the average duration of seasonal influenza was 11 weeks (range 4.9–16.9 weeks). The duration for 2023/24 season was unusually long at 32.9 weeks. Coinfection with influenza was identified in 17.2% (range, 10.2%−22.9%) of pneumococcal hospitalizations during the seasonal periods, compared to 6.0% during the non-seasonal period, and the difference was statistically significant across the baseline, pandemic and post-pandemic periods. The total proportion of influenza coinfection in post-pandemic period (14.3%) was higher than those in the baseline (11.5%) and pandemic periods (3.2%) (*P <*0.001). During the pandemic period, influenza coinfections were only observed between January and March 2020, with no instances detected from April 2020 to December 2022 (Fig. 3A).

**Table 2:** Summary of influenza seasons during 2015–2024, Hong Kong

Influenza season	Starts date	End date	Onset season^a^	Duration in weeks	Circulating subtype(s)	% coinfection (95% confidence interval)
2014/15	01-Jan-15	11-Apr-15	Winter	14.3	H3	18.9 (12.4–27.5)
2015	24-May-15	25-Jul-15	Spring	8.9	H3	12.9 (5.6–25.4)
2015/16	31-Jan-16	07-May-16	Winter	13.9	H1, B	14.6 (9.3–22.0)
2016	11-Sep-16	15-Oct-16	Autumn	4.9	H3	22.9 (11.4–41.0)
2017	23-Apr-17	19-Aug-17	Spring	16.9	H3	22.2 (15.4–30.8)
2017/18	24-Dec-17	24-Mar-18	Winter	12.9	B	20.1 (15.0–26.3)
2018/19	09-Dec-18	06-Apr-19	Winter	16.9	H1	18.4 (13.2–24.9)
2019	16-Jun-19	20-Jul-19	Summer	4.9	H1, H3, B	10.2 (3.3–23.8)
2019/20	22-Dec-19	01-Feb-20	Winter	5.9	H1	13.4 (7.1–22.9)
2023	26-Mar-23	20-May-23	Spring	7.9	H1	20.7 (12.3–32.7)
2023	02-Jul-23	28-Oct-23	Summer	16.9	H3	16.3 (11.2–23.1)
2023/24	10-Dec-23	27-Jul-24	Winter	32.9	H3, H1	14.5 (11.0–18.7)

^a^Spring, March–May; summer, June-Aug; autumn, September–November; winter, December-February

In the pandemic and post-pandemic periods, 72.7% and 88.2% of cases were tested for COVID-19, respectively (Figure S4). From January 2020 to January 2022, there were minimal occurrences of COVID-19 coinfections (Fig. 3A). Notable spikes in coinfection rates, reaching 71.2% and 33.3%, were observed during two COVID-19 Omicron waves in February-April 2022 and October 2022-February 2023, respectively. In the post-pandemic period from March 2023 onward, COVID-19 coinfections were detected regularly with an average monthly prevalence of 12.4% (range, 3.2%−30.6%).

Correlation analyses were conducted to investigate the impact of changes in viral activity on monthly pneumococcal-viral coinfections. The results revealed a strong positive correlation between indicator of influenza activity and monthly pneumococcal hospitalizations (Spearman’s rank correlation coefficient = 0.755, Fig. 3A and Figure S5), whereas the correlation with COVID-19 indicator was weak (Spearman’s rank correlation coefficient = 0.374, Fig. 3A and Figure S5). Strong positive correlations were also observed between indicator of influenza activity and influenza coinfections (Spearman’s rank correlation coefficient = 0.876, Fig. 3B and Figure S5), as well as between indicator of COVID-19 activity and COVID-19 coinfections (Spearman’s rank correlation coefficient = 0.847, Fig. 3C and Figure S5).

### Changes in patient outcomes by time periods

**Fig. 4:**
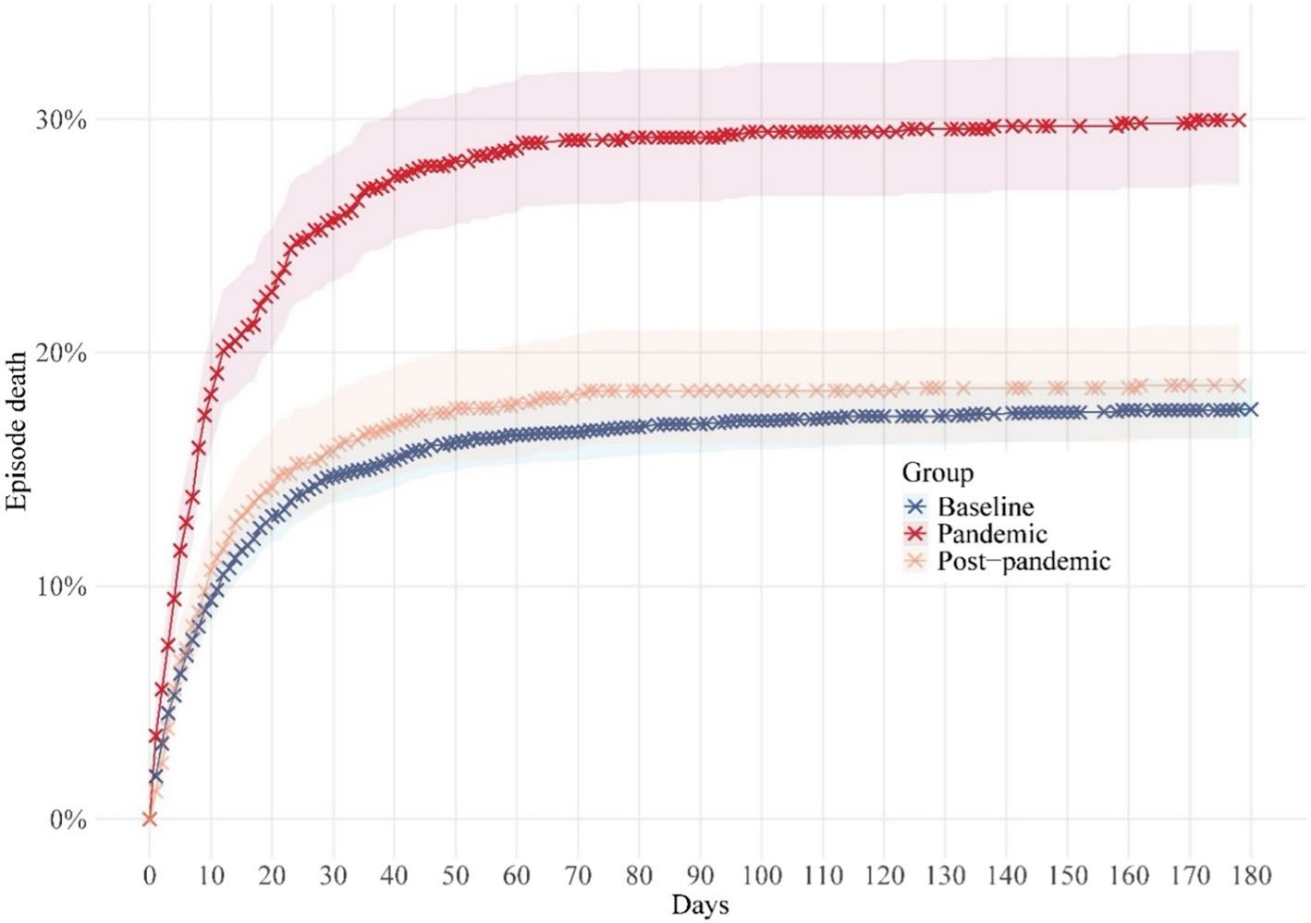
Kaplan–Meier curves for cumulative episode death for pneumococcal hospitalizations in baseline(2015–2019), pandemic(2020–2022), and post-pandemic(2023–2024) periods. The log-rank test comparing baseline versus pandemic and baseline versus post-pandemic periods resulted in *p*-values of < 0.001 and 0.056, respectively. Colour bands show 95% confidence intervals

The cumulative in-patient mortality was significantly higher during the pandemic period than in the baseline period (Fig. 4). The two curves exhibited a divergence and eventually reached plateaus approximately 60 days after admission. In the post-pandemic period, in-patient mortality returned to baseline level.

The median length of stay was 9 ± 14 days in both baseline and pandemic period but was decreased to 8 ± 12 days in the post pandemic period (Table 3). In the baseline period, the proportion of patients requiring IMV was higher at 15.3% compared to the pandemic period (11.4%) and post-pandemic period (8.3%). The proportion of patients admitted to the ICU during the pandemic period was 10.9%, comparing to 12.5% in the baseline period and 14.0% in the post-pandemic period. The mortality rate in the pandemic period was higher at 30.2% compared to 18.1% in the baseline period and 18.8% in the post-pandemic period. Age-related variations were observed in the rates of patients requiring IMV, ICU admission, and having episode deaths. Patients aged 18–59 years had the higher proportions of IMV and ICU admission, while patients aged 70 years or older had the highest proportion of episode deaths.

**Table 3:** Outcome of patients with pneumococcal infections in three time periods

Outcome	Baseline (*n* = 3495)	Pandemic (*n* = 1014)	Post-pandemic (*n* = 1008)	*P* value
LOS, median ± IQR	9 ± 14	9 ± 14	8 ± 12	0.008
IMV, % (no.)				
18–59 years	17.5 (149)	23.8 (30)	14.0 (25)	0.083
60–69 years	16.9 (136)	18.7 (26)	13.8 (22)	0.492
≥ 70 years	13.7 (252)	8.0 (60)	5.5 (37)	< 0.001
Total	15.3 (537)	11.4 (116)	8.3 (84)	< 0.001
ICU admission, % (no.)				
18–59 years	17.6 (150)	27.0 (34)	25.1 (45)	0.007
60–69 years	14.4 (116)	23.7 (33)	26.3 (42)	< 0.001
≥ 70 years	9.3 (171)	5.9 (44)	8.1 (54)	0.015
Total	12.5 (437)	10.9 (111)	14.0 (141)	0.118
Episode death, % (no.)				
18–59 years	9.5 (81)	18.3 (23)	7.3 (13)	0.004
60–69 years	14.4 (116)	19.4 (27)	13.8 (22)	0.275
≥ 70 years	23.8 (437)	34.2 (256)	23.2 (155)	< 0.001
Total	18.1 (634)	30.2 (306)	18.8 (190)	< 0.001

*Abbreviations*: *ICU* intensive care unit, *LOS* length of hospital stay

**Fig. 5:**
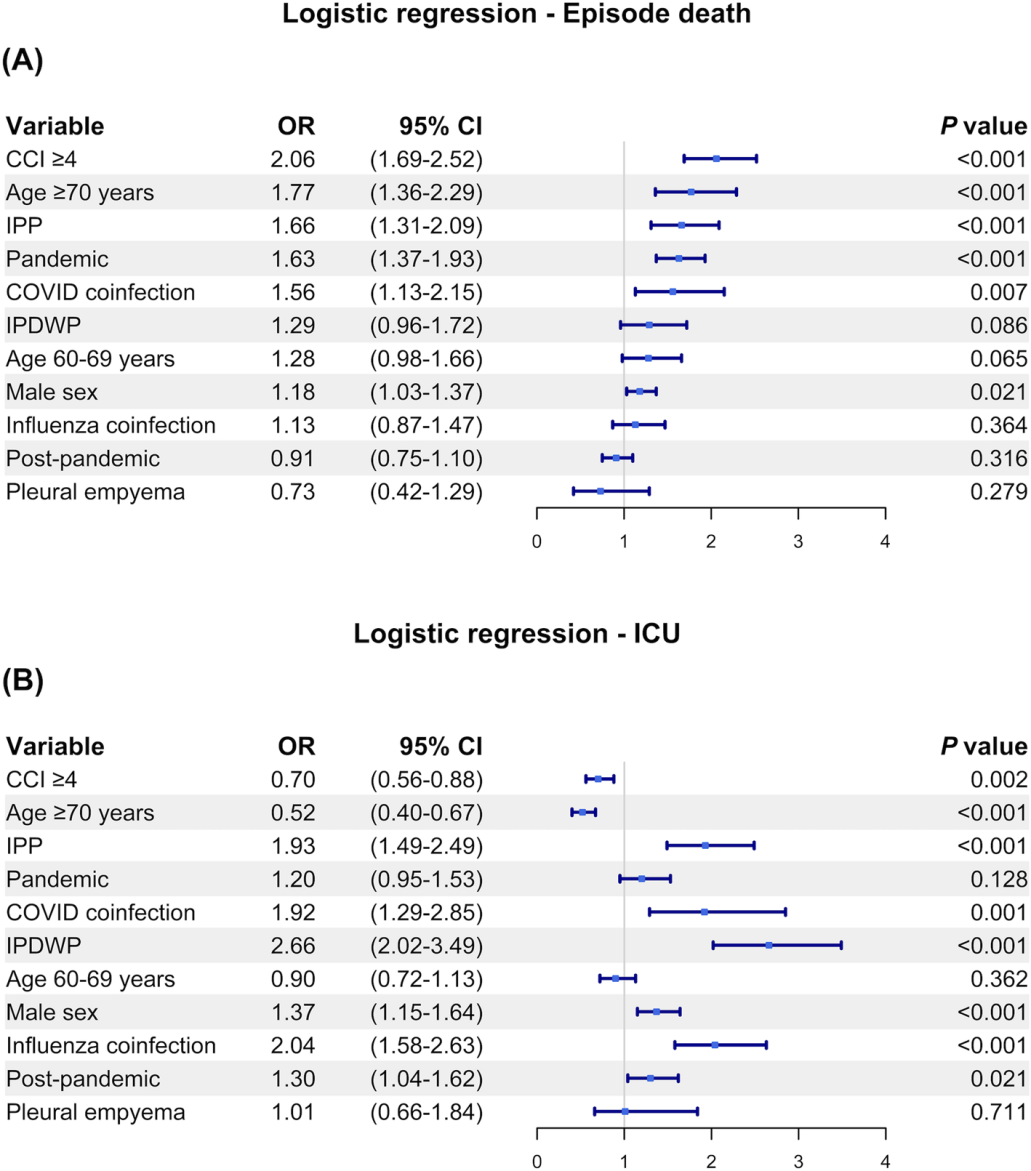
Forrest plot of multivariate anlaysis for risk factors associated with adverse outcome in pneumococcal hospitalizations. **A** Episode death, (**B**) Intensive care unit (ICU) admission

Predictive variables for adverse clinical outcomes from multivariate analysis are summarized in Fig. 5. A higher CCI ≥ 4 (OR 2.06), old age ≥ 70 years (OR 1.77), IPP (OR 1.66), hospitalization during pandemic period (OR 1.63) and COVID-19 coinfection (OR 1.56) were significantly associated with higher likelihood of episode death. IPP (OR 1.93), COVID-19 coinfection (OR 1.92), IPWWP (OR 2.66), male sex (OR 1.37), influenza coinfection (OR 2.04) were significantly associated with a higher likelihood of ICU admission. The sensitivity analyses using Robust Poisson regression and Log-binomial regression produced similar results to the primary analysis (Figure S6).

## Discussion

This territory-wide study described the changes in pneumococcal disease incidence, coinfection with influenza and COVIID-19, and clinical outcomes before, during and after the COVID-19 pandemic. This is a continuum of our previous study which examined the incidence and severity of pneumococcal disease during the early phase of the COVID-19 pandemic [15]. Previous studies have reported a rebound in the number of IPD cases in Canada, Germany, Switzerland, and the United Kingdom shortly after the lifting of public health and social measures during 2021–2023, with incidence reaching or surpassing pre-COVID-19 pandemic levels [27–30]. In this study, we present data on both pneumococcal pneumonia and IPD, indicating that both forms of the disease have returned to levels similar to the baseline period, but the rebound occurred at a slower pace than previously observed [27–30]. This slower rebound is likely due to a significant portion of the local population continuing to wear masks in public places. A randomized controlled study demonstrated a reduction in self-reported respiratory symptoms through masking in public areas, suggesting that this could be a strategy in preventing pneumococcal disease [27].

In this study, the overall disease burden of NIPP was found to be five times greater than that of IPP, with the ratio increasing from 2020 onwards. This temporal increase can be attributed to the improved identification of pneumococcal etiology from the increased availability of the UAT during and after the COVID-19 pandemic. In adults, the UAT is a method with good sensitivity and excellent specificity for diagnosis of pneumococcal pneumonia [31]. A meta-analysis estimated the non-invasive to invasive pneumococcal pneumonia ratio to be around 3:1, with the UAT detecting an additional 11% of cases as pneumococcal beyond traditional methods [32]. The ratios of NIPP to IPP in our study indicates that UAT could potentially diagnose an even larger proportion of pneumonia cases as pneumococcal. Although the commercially available immunochromatographic UAT can detect all serotypes, its sensitivity is lower than that of the noncommercial Luminex technology-based multiplex serotype-specific urinary antigen detection method [33]. The actual burden of pneumonia associated with *Streptococcus pneumoniae* is likely underestimated.

This study demonstrated that coinfection with influenza or COVID-19 was common among patients with pneumococcal disease and associated with worse outcomes. Our study utilized real-world data with positive virology results, while previous studies on pneumococcal and influenza coinfection relied on national or regional surveillance data and mathematical modeling [34–36]. Despite seasonal variability, our study found higher proportions of influenza coinfection in the baseline and post-pandemic periods, compared to the 5%−6% reported in the United states during 1995–2006 [35]. In a study of 271 Alaska patients with laboratory-confirmed IPD during the pandemic, 20% had COVID-19 coinfection, resulting in higher mortality rates compared to those without [37]. The present study uncovered a notable rise in COVID-19 coinfection during the pandemic waves and its sustained prevalence post-pandemic, highlighting its correlation with adverse outcomes. Additionally, a comparative analysis was conducted regarding influenza coinfection. These findings underscore the importance of administering dual influenza and COVID-19 vaccinations to individuals at risk of pneumococcal infection. This recommendation is particularly crucial for individuals aged 70 years and above, who constitute a large portion of pneumococcal hospitalizations and mortalities. This aligns with the current local vaccination program, which recommends individuals aged 50 and above receive the influenza vaccine and the COVID-19 vaccine annually, and that individuals aged 65 years old and above receive pneumococcal vaccines [6].

The serotype distribution analysis conducted in our study revealed that the prevalence of serotypes remained unchanged across the three periods. This is consistent with recent studies from Europe and South Africa, which demonstrated similar prevalence of the major pneumococcal serotypes responsible for nasopharyngeal colonization and disease [38–40]. Despite the widespread use of PCV13 in our locality, the emergence and persistence of serotype 3 as the most prevalent serotype is concerning. Previous observations have shown that PCV13 has not had an impact on the incidence of serotype 3 IPD [8, 22]. In a global surveillance analysis of more than 500,000 IPD cases, serotype 3 has been identified as a top-ranking serotype in children under 5 years old and adults over 50 years old at both PCV10 and PCV13 vaccination sites [41, 42]. The similar patterns observed at PCV10 and PCV13 sites suggest that PCV13 does not protect against serotype 3 [41]. Furthermore, PCVs containing serotype 3 have not elicited adequate antibody responses needed to provide protection against this particular serotype [43–46]. A different technology than the one used in PCV13 is required to provide protection against serotype 3 [41, 42].

A high comorbidity score and advanced age were associated with reduced ICU admissions, aligning with the local policy that excludes patients with end-stage renal failure, advanced malignancy, and dementia from ICU admission triage [47].

### Strengths and limitations

A strength of this study is the utilization of both pneumococcal ICD-9 coding and microbiological results to identify cases, allowing for the detection of a greater number of pneumococcal hospitalizations compared to relying solely on ICD-9 coding, as was the case in our previous study [15]. As a territory-wide study, the findings provide a more representative portrayal of the epidemiology of pneumococcal disease in this region. Another strength lies in the utilization of real-world virological results to explore temporal variations in coinfection frequencies of influenza and COVID-19, followed by a comparison of the outcomes, which was not undertaken in previous studies [34–37].

The study has limitations. While subjects with influenza or COVID-19 coinfection were examined, the frequencies of testing were not even across the three time periods, potentially leading to an underestimation of the coinfection rate. Additionally, serotype results were only available for a small proportion of cases, precluding the analysis of clinical outcomes by serotypes. Moreover, the pneumococcal vaccination status was not included in this cohort and is an important factor that might affect the presentation and outcomes of pneumococcal disease. Finally, the database contains microbiological results exclusively from public hospitals. Microbiological results from private healthcare facilities before hospitalization and tests sent to the Department of Health were not accessible, which could account for the lack of pneumococcal results in some cases with pneumococcal coding. ICD-9 coding was carried out by trained staff, minimizing the risk of confounding from miscoding, which should be small and consistent throughout all three time periods.

## Conclusions

In summary, our study showcased the influence of COVID-19 on pneumococcal disease epidemiology during and after the pandemic. It highlighted the adverse effect of coinfections involving influenza and COVID-19 on the outcomes of patients with pneumococcal disease. This underscores the importance of vaccinating vulnerable populations against pneumococcal, influenza, and COVID-19 infections.

## Supplementary Information

Supplementary Material 1

## Data Availability

No datasets were generated or analysed during the current study.

## Abbreviations

COVID-19: Coronavirus Disease 2019
IPP: Invasive pneumococcal pneumonia
IPDWP: Invasive pneumococcal disease without pneumonia
NIPP: Non-invasive pneumococcal pneumonia
IPD: Invasive pneumococcal disease
SARS-CoV-2: Severe acute respiratory syndrome coronavirus 2
CDARS: Clinical Data Analysis and Reporting System
CHP: Centre for Health Protection
UAT: Pneumococcal urinary antigen test
ICU: Intensive care unit
IMV: Invasive mechanical ventilation
