# Supplementary material for: Changes in the incidence, viral coinfection pattern and outcomes of pneumococcal hospitalizations during and after the COVID-19 pandemic
Source: Pneumonia (Nathan). 2025 Apr 25;17:9. doi: 10.1186/s41479-025-00164-0 (PMC12023597; doi:10.1186/s41479-025-00164-0)

Supplementary file

Figure S1. Flowchart of patient selection in this study

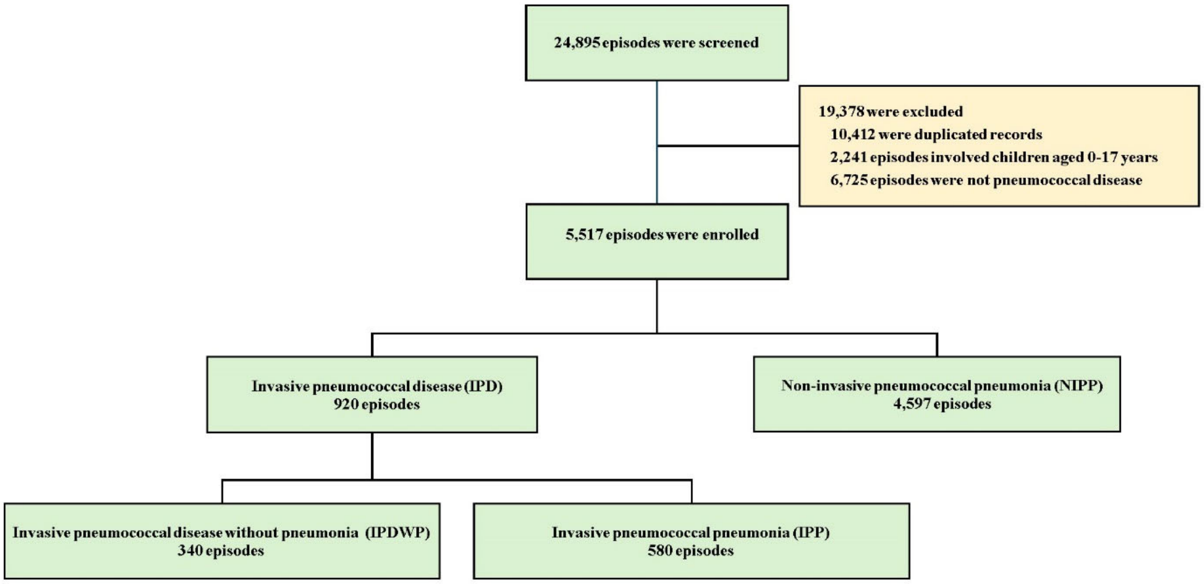

Figure S2. Temporal changes in number and incidence rate of pneumococcal hospitalizations by data acquisition source. "Laboratory-confirmed", cases with positive pneumococcal test results; "ICD coding", cases identified by pneumococcal ICD-9 codes without positive test results.

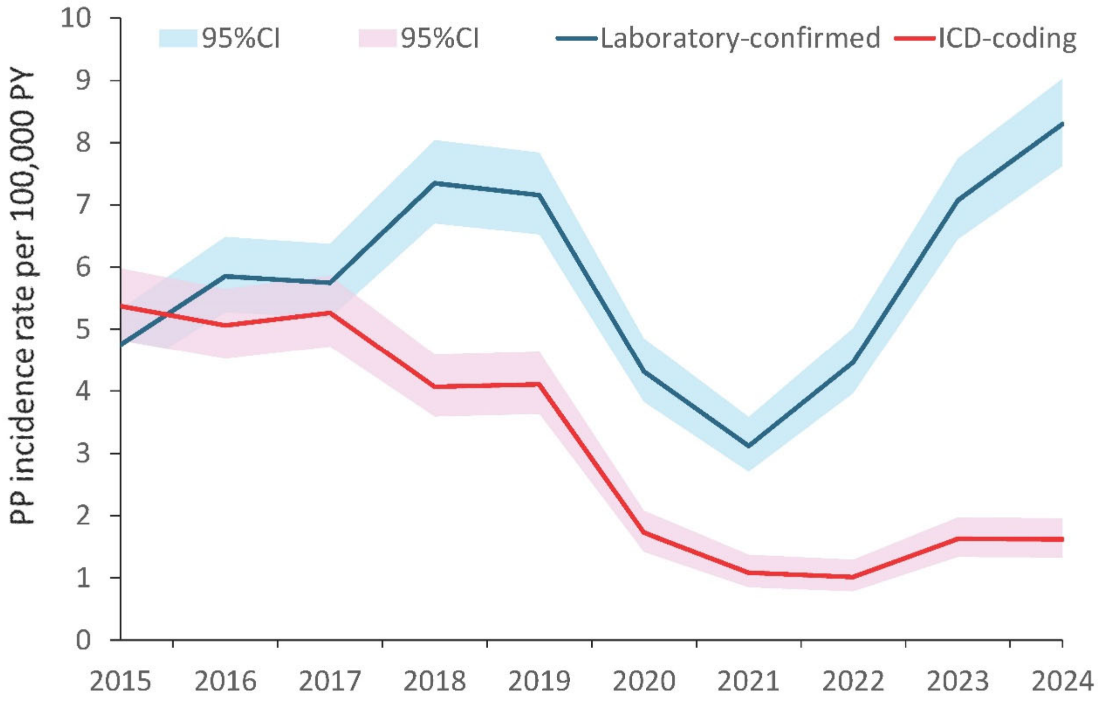

Figure S3. Occurrence of influenza coinfection among patients hospitalized for pneumococcal disease in Hong Kong

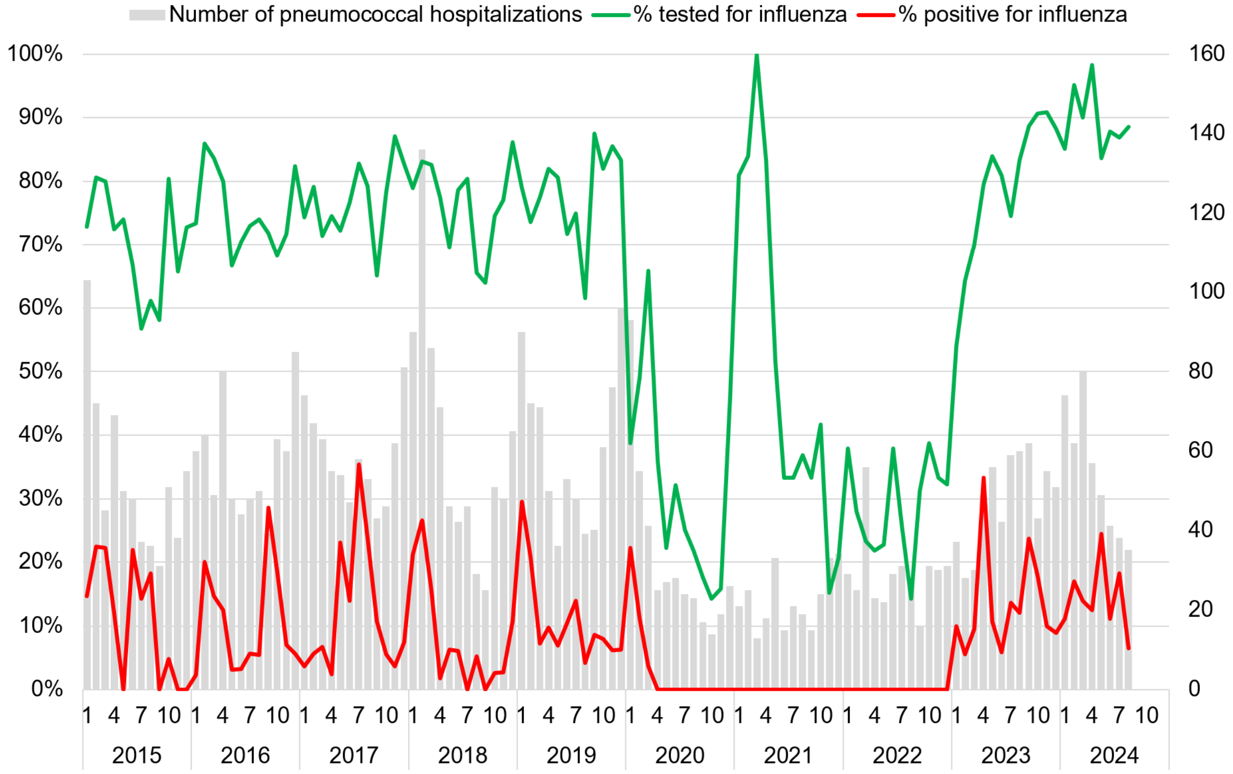

Figure S4. Occurrence of COVID-19 coinfection among patients hospitalized for pneumococcal disease in Hong Kong [a]

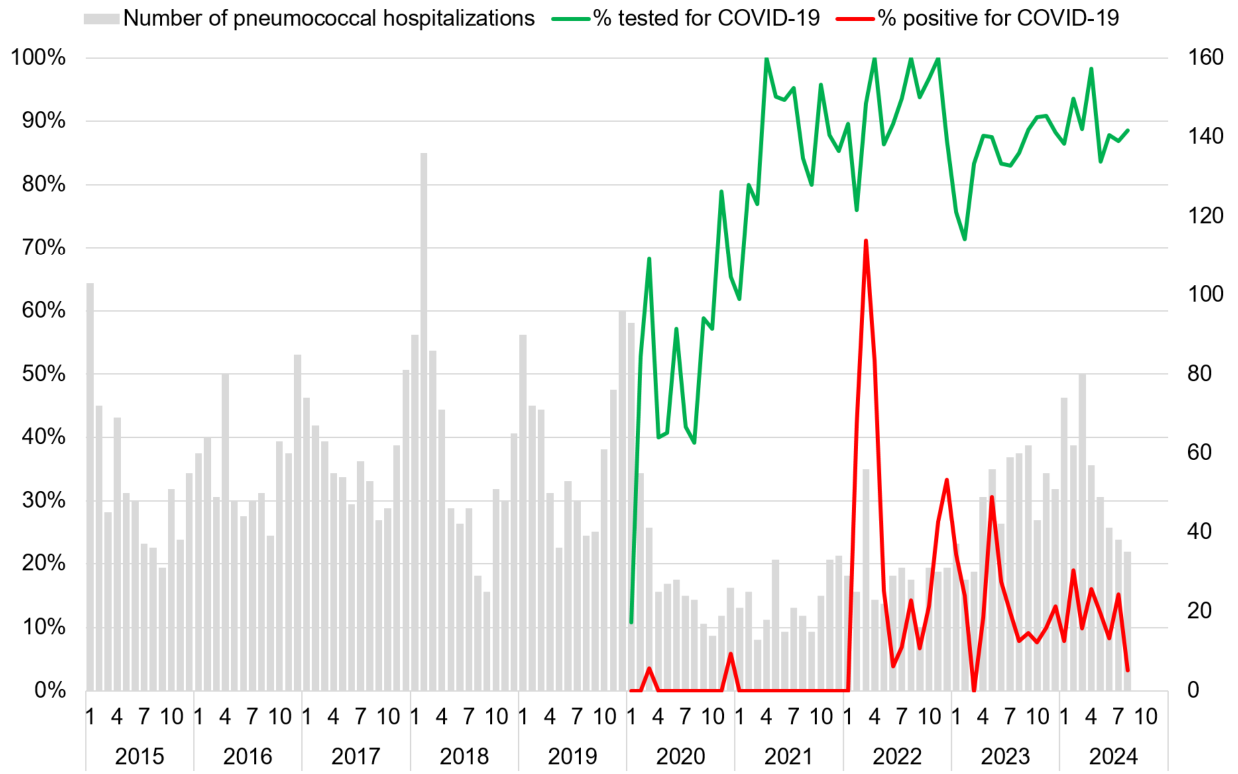

[a]Hong Kong implemented strigent measures for control of COVID-19 between January 2020 and December 2022. In Hong Kong, five waves of COVID-19 occurred between January 2020 and December 2022, including first wave, 23 January 2020 to 14 March 2020 with a total of 142 COVID-19 cases; second wave, 14 March 2020 to 30 June 2020 with a total of 1064 COVID-19 cases; third wave, 1 July 2020 to 31 October 2020 with a total of 4118 cases; fourth wave, 1 November 2020 to 30 April 2021 with a total of 6451 COVID-19 cases; and fifth wave (Omicron), 31 December 2021 to 31 December 2022 with more than 1.7 million COVID-19 cases.

Figure S5. Correlation analyses. (A) Pneumococcal hospitalization and influenza activity indicator, (B) pneumococcal hospitalization and COVID-19 activity indicator, (C) Coinfection with influenza and influenza activity indicator, (D) Coinfection with COVID-19 and COVID-19 activity indicator. In the graphs, each colored dot represented the data for one month during the study period, with data points colored according to the season of the month.

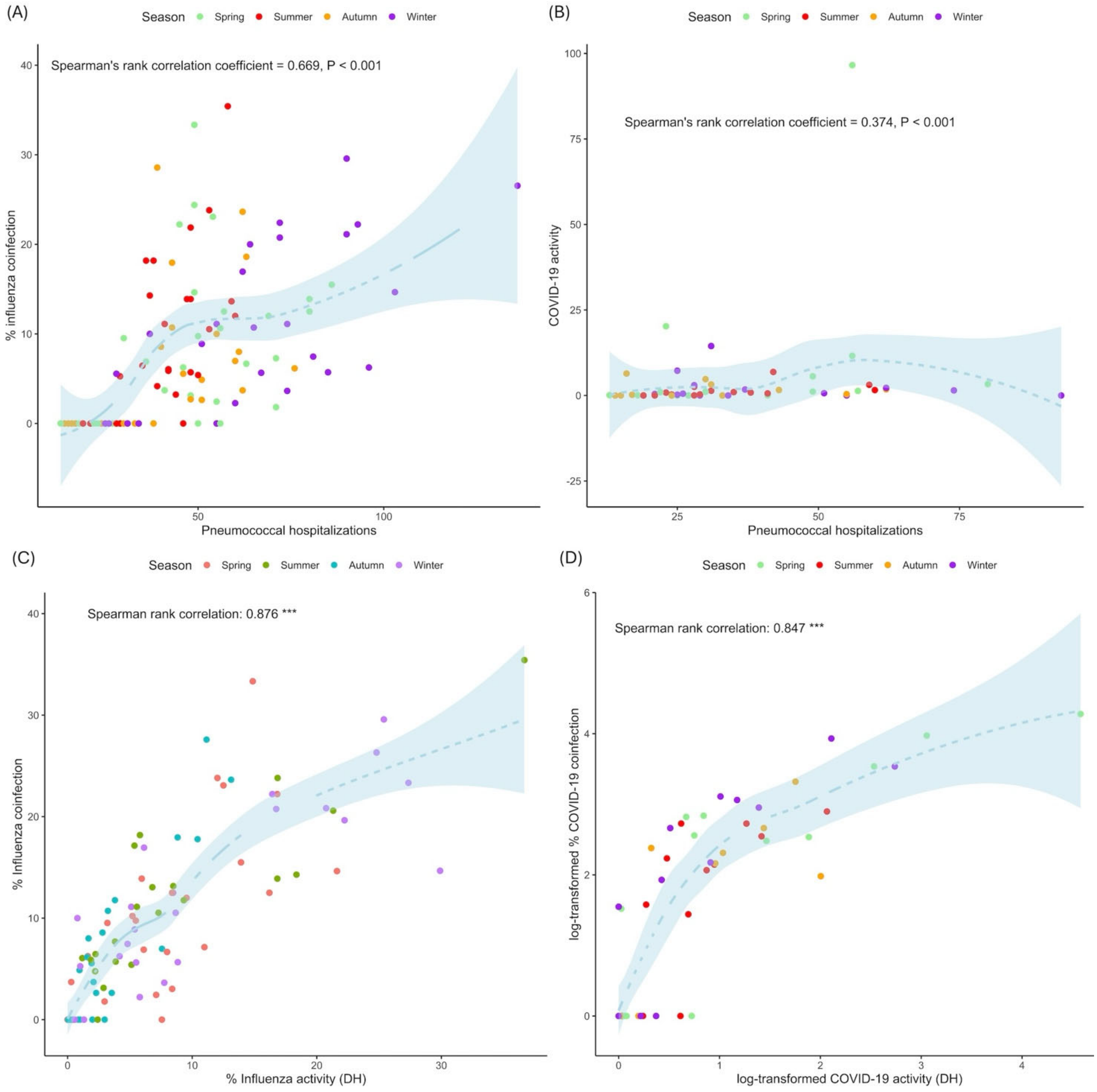

Figure S6. Forrest plot of two sensitivity analyses for risk factors associated with adverse outcome in pneumococcal hospitalization. (A) Episode death, Robust Poisson regression (B) ICU admission, Robust Poisson regression, (C) Episode death, Log-binomial regression (D) ICU admission, Log-binomial regression

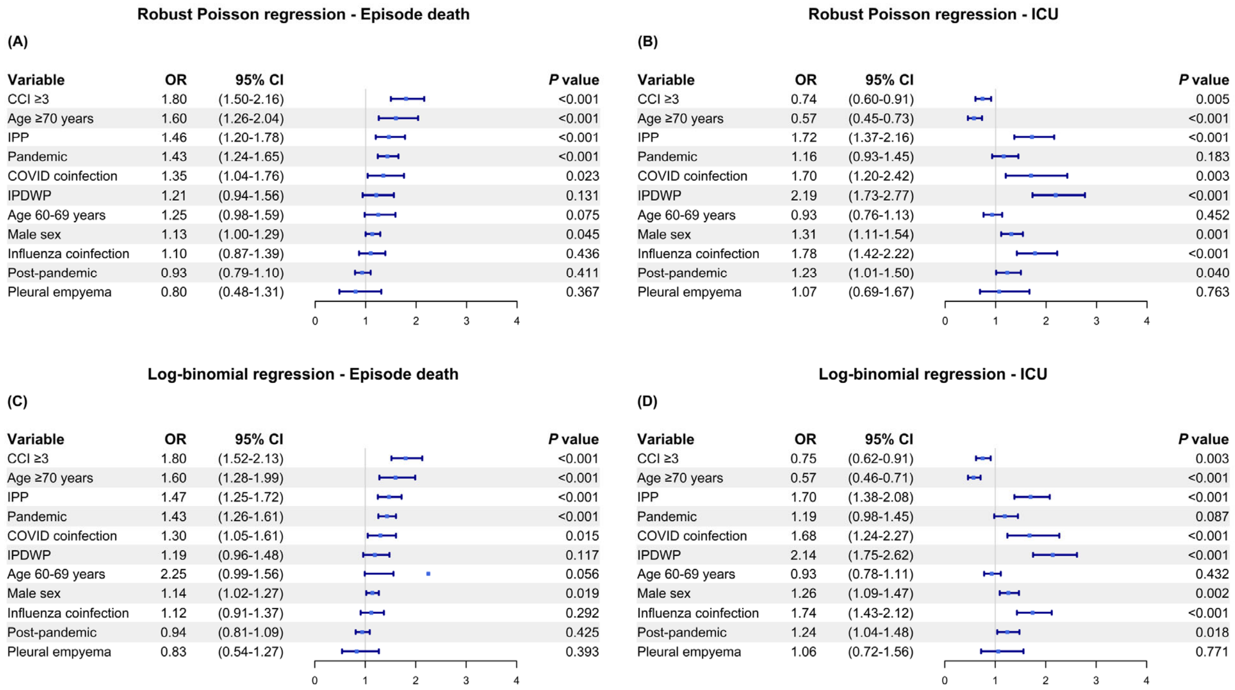

Supplement: Supplementary file 1 — Supplementary Material 1 [file 41479_2025_164_MOESM1_ESM.pdf]
